# Current status on lithium disilicate and zirconia: a narrative review

**DOI:** 10.1186/s12903-019-0838-x

**Published:** 2019-07-04

**Authors:** Fernando Zarone, Maria Irene Di Mauro, Pietro Ausiello, Gennaro Ruggiero, Roberto Sorrentino

**Affiliations:** Department of Neurosciences, Reproductive and Odontostomatological Sciences, University “Federico II” of Naples, Viale Pansini, 5 -, 80131 Naples, Italy

**Keywords:** Lithium disilicate, Zirconia, ZLS, Ceramic, Minimally invasive, E.max, MDP, Aging, Translucent cubic zirconia

## Abstract

**Background:**

The introduction of the new generation of particle-filled and high strength ceramics, hybrid composites and technopolymers in the last decade has offered an extensive palette of dental materials broadening the clinical indications in fixed prosthodontics, in the light of minimally invasive dentistry dictates. Moreover, last years have seen a dramatic increase in the patients’ demand for non-metallic materials, sometimes induced by metal-phobia or alleged allergies. Therefore, the attention of scientific research has been progressively focusing on such materials, particularly on lithium disilicate and zirconia, in order to shed light on properties, indications and limitations of the new protagonists of the prosthetic scene.

**Methods:**

This article is aimed at providing a narrative review regarding the state-of-the-art in the field of these popular ceramic materials, as to their physical-chemical, mechanical and optical properties, as well as to the proper dental applications, by means of scientific literature analysis and with reference to the authors’ clinical experience.

**Results:**

A huge amount of data, sometimes conflicting, is available today. Both in vitro and in vivo studies pointed out the outstanding peculiarities of lithium disilicate and zirconia: unparalleled optical and esthetic properties, together with high biocompatibility, high mechanical resistance, reduced thickness and favorable wear behavior have been increasingly orientating the clinicians’ choice toward such ceramics.

**Conclusions:**

The noticeable properties and versatility make lithium disilicate and zirconia materials of choice for modern prosthetic dentistry, requiring high esthetic and mechanical performances combined with a minimal invasive approach, so that the utilization of such metal-free ceramics has become more and more widespread over time.

## Background

At “The Digital Dentistry Society II Consensus Conference on Digital Technologies – Marrakech 2018” the main topics of digital interest were thoroughly discussed, in order to draw clinical recommendations based on scientific evidence and, when missing, on the clinical experience shared by the scientific community. The present narrative review is focused on the technical and clinical profile of the two most popular metal-free materials, lithium disilicate and zirconia, in order to briefly shed light on their different indications, advantages and shortcomings.

## Methods

An extensive research has been carried out in the literature available on the subject, worldwide, limiting itself exclusively to articles in english, available on the main search engines (Pubmed, Embase, Scopus) and published in the most important indexed journals of the Materials and Dental sector, with and without impact factor. The results highlighted in this narrative review were extrapolated from this literature search, with reference to the authors’ clinical experience.

## Results

### Lithium disilicate

#### Physico-chemical features, optical and mechanical properties

Lithium disilicate (LS_2_) is classified as a glass-ceramic, in the class of particle-filled glass materials. Introduced on the market in the 90s with the commercial formulation named “IPS Empress 2” (Ivoclar Vivadent, Schaan, Liechtenstein), it was composed of 65 vol% lithium disilicate, small needle-shaped crystals (3–6 μm × 0.8 μm) embedded in a glass matrix, with a 1 vol% porosity [1–3], showing valuable mechanical characteristics (flexural strength: 350 MPa; fracture toughness (KIC): 3.3 MPa√m; heat extrusion temperature: 920 °C; thermal expansion coefficient (CTE): 10.6 + 0.25 ppm/°C). At first, this material was made commercially available as ingots, to be utilized according to the “heat-pressing” fabrication procedure, similar to the classic “lost wax” technique for metal-alloy casts, aimed at producing cores, hot pressed into a mold. In order to get an appealing reproduction of the optical characteristics of natural teeth, the cores are lately veneered with a very translucent fluorapatite ceramic, containing 19–23% of fluorapatite crystals (Ca_5_(PO_4_)_3_F) embedded in a glassy matrix [4].

Thanks to an optimization of the processing parameters, allowing the formation of smaller and more uniformly distributed crystals, in 2005 a new formulation of LS_2_ was marketed as “IPS e.max Press” (Ivoclar Vivadent), exhibiting improved mechanical properties and optical features (flexural strength: 370–460 MPa; fracture toughness (KIC): 2.8–3.5 MPa√m), much higher than the older glass-ceramics. The high mechanical performance of this material is due, on one side, to a layered, tightly interlocked distribution of the elongated disilicate crystals, hindering crack propagation across the planes and, on the other side, to a mismatch between the thermal expansion coefficients of LS_2_ crystals and the glassy matrix, so that the latter induces a tangential, compressive stress around the crystals [2]. Besides the production of ceramic cores for bilayered crowns, the increase of strength and toughness of IPS e.max Press has allowed to extend its clinical indication to monolithic restorations, without veneering ceramic, anatomically shaped, colored by surface stains and characterized by a higher fatigue resistance than the bilayered ones.

Besides the heat-pressed technique, the widespread, increasing implementation of computer-aided design/computer-aided manufacturing (CAD-CAM) technologies has led to the introduction of ceramic blocks aimed at the production of restorations by milling devices (IPS e.max CAD), also suitable for chairside production of restorations. Partially, pre-crystallized blocks are manufactured in a “blue state”, containing 40% of metasilicates (Li_2_SiO_3_) in addition to lithium disilicate crystal nuclei (Li_2_Si_2_O_5_). Such blocks are characterized by moderate flexural strength of ~ 130 MPa, resulting in higher cutting efficiency, easier and faster workability and lower wear of the milling tools [2, 3, 5]. The milling procedure is performed in this pre-crystallized state and, after its completion, it is followed by a heating cycle (840°-850 °C for 10 min) that turns metasilicate crystals into lithium disilicate (~ 70%), increasing the flexural strength up to values of 262 ± 88 MPa, together with a fracture toughness of 2.5 MPa·m^1/2^. The blocks are available in different colors, obtained by dispersing staining ions in the glassy matrix [6] and in different degrees of translucency, on the basis of the size and distribution of the crystals in the glassy matrix [4]. The variability of flexural strength of lithium disilicate among heat-pressed and CAD-CAM blocks with different translucency is still under debate [7, 8]. Particularly, the flexural strength of IPS e.max Press and IPS e.max CAD was reported to be similar and the manufacturing process did not seem to affect the mechanical characteristics of lithium disilicate ceramics; moreover, the flexural strength was significantly influenced by translucency only for CAD-processed materials [7].

In vitro fully anatomical e.max CAD crowns have been shown to exhibit fracture resistance that is suitable for posterior, monolithic restorations [9] and to be more resistant to fatigue in cyclic loading than veneered zirconia, that is more prone to chipping [10]. For the high interest generated by its clinical versatility, further developments are expected on this material, being it influenced by different production processes, like thermal gradients, times and rates, that affect its microstructure and mechanical properties. It has been shown, for instance, that extending temperature range (750–840 °C, compared to the standard 820–840 °C) or prolonging holding time (14 min vs 7 min at 840 °C) increase elastic modulus and hardness properties, without affecting flexural strength and fracture toughness [11]. Moreover, new technologies, as spark plasma sintering, can induce a refinement and a densification of the nano-crystalline microstructure, increasing lithium disilicate and metasilicate phases and reducing lithium orthophosphate and cristobalite/quartz phases [12, 13].

As regards mechanical resistance, it has been clearly demonstrated that, in vitro, veneered LS_2_ crowns exhibit significantly lower fracture load values (1431.1 ± 404.3 N) compared to monolithic ones (2665.4 ± 759.2 N), the main failure mechanism being bulk fracture initiating from the occlusal surface [14]. To date, there is strong evidence from in vitro studies that, differently from bilayered restorations, monolithic ones show fracture strength and fatigue resistance suitable for use in the posterior areas, both in tooth- and implant-supported single crowns (SC) and 3-unit fixed dental prostheses (FDPs) [15–22].

Monolithic LS_2_, as well as Zirconia reinforced-Lithium Silicate ceramics (ZLS), offers higher fracture resistance than bilayered, hand-veneered zirconia [20], while a recent in vitro research has shown that load-to-fracture values of monolithic zirconia are higher than those of LS_2_; the latter, in turn, are higher than those of ZLS [23].

It has to be pointed out, however, that, particularly as regards LS_2_, fatigue resistance is strongly influenced by many experimental variables, like amount of cyclic loading, abutment and antagonist design and material, thermocycling parameters and test environment; for this reason, the heterogeneity and lack of standardization in research designs, tested materials and experimental conditions make a comparison of data not easily feasible [24].

#### Abrasiveness and wear

As to wear and abrasiveness, LS_2_ shows quite favourable properties, that are highly depending on the surface characteristics of the restoration. When accurately polished at its surface, the material exhibits convenient tribological behaviour in vitro, in terms of friction and wear of restorations, being its abrasiveness quite close to enamel, although more aggressive when compared to type III gold [25] or to polished monolithic zirconia in in vitro simulations [26–28]. Such favourable wear behaviour and durability have been also confirmed by some in vivo evidence [15].

On the other hand, it has been reported that grinding, glaze coating and fluorapatite ceramic veneering can increase wear, both of the antagonist teeth and of the restoration itself; at the same time, surface roughness can also be increased, besides a reduction of gloss, in the presence of basic pH environment and after toothbrushing with abrasive toothpaste [29–33]. For these reasons, when it is not crucially needed for esthetic reasons, glazing of monolithic restorations should be avoided on the occlusal surfaces in posterior sites and only limited to the esthetically relevant zones; moreover, careful polishing procedures should always follow any occlusal grinding or esthetic refinement of disilicate restorations, although in vitro evidences at scanning electron microscope (SEM) have shown that LS_2_ is one of the most critical materials to adjust intraorally, due to significant chip accumulation in the diamond burs, requiring higher machining forces and energy, with likely onset of intergranular and transgranular fractures, besides risks of thermal damage to tissues and restorations [32].

#### Biocompatibility

One of the strongest points of LS_2_ is the excellent quality of soft tissue response. In vitro, this material exhibits high levels of biocompatibility, not only due to low plaque retention, but also to adhesion and proliferation of human epithelial cells [34] and human gingival fibroblasts [35], particularly when its surface is polished. In vivo, in the presence of LS_2_ restorations no inflammatory reactions were detected, analyzing the concentration of inflammation indicators in the gingival crevicular fluid; the same results were found with zirconia restorations [36]. Such favourable tissue responses have also been confirmed by tissue culture data [34]. In clinical experience, LS_2_ restorations are likely to yield very natural and sound aspect of soft tissues when in contact with marginal gingiva or peri-implant mucosa, in the presence of subgingival margins.

#### Surface treatment and cementation

In addition to excellent biocompatibility and high mechanical properties, LS_2_ exhibits very good esthetic features, especially as regards translucency, that is about 30% higher than conventional zirconia [37]. Moreover, for the presence of silica, LS_2_ is an acid-sensitive ceramics, so that high strength of adhesion to the substrate is expected, due to both micromechanical and chemical bonding mechanisms. Micromechanical interlocking between ceramics and resin cement at the intaglio surface is based on the creation of surface microirregularities, pits and roughness by means of acid etching and/or physical treatments like alumina particles sandblasting or diamond bur grinding. For the glass-ceramic class, to date hydrofluoric acid (HF) etching is the best-established procedure, to be performed according to validated protocols taking into account both acid concentration and etching time. For LS_2_, 20 s HF etching (at 5% concentration) is suggested, that is a shorter time than requested for feldspathic and leucite-based ceramics (generally 60 s). Higher HF concentrations (9–10%) and longer etching times have been shown to be too aggressive and can introduce relevant damages, not only to the surface but also to the internal microstructure of the material, negatively influencing mechanical performance (reduction of flexure strength), adhesion potential and long-term success of ceramic restorations, particularly when thickness is low [38–41]. Another system to create surface microirregularities is sandblasting LS_2_ with aluminum oxide particles. Nevertheless, it has been shown that this procedure, as well as laser etching, can determine excessive loss of material, with surface modifications that are less uniformly distributed than after HF etching and that can significantly reduce flexural strength [42, 43]. In addition to micromechanical interlocking, as for all silica-based materials, adhesive bonding of LS_2_ is efficiently increased by silane, ensuring a chemical interaction between the resin-based agent and the ceramics, obtained forming strong siloxane linkages [44–50].

Recently, it has been shown that the use of silane combined to a phosphate functional monomer, the 10-Methacryloyloxydecyl-Dihydrogen-Phosphate (10-MDP), creating an acidic environment further improves the bond strength of resin-based luting cement to lithium disilicate ceramics [51].

#### Clinical indications and performances

As regards clinical indications of LS_2_, it has to be pointed out that this is one of the most versatile metal-free materials for its high esthetic potential, good mechanical properties and favourable bonding strength to dental tissues, thanks to its silica content. Lithium disilicate ceramics can be utilized both for tooth- and implant-supported restorations, ranging from SCs to FDPs, from anterior veneers to posterior inlays, onlays and overlays [4, 7].

To date, due to its relatively recent market introduction, there is still a lack of data about long-term outcomes of LS_2_ restorations, particularly as regards CAD-CAM production. Prospective, medium-term studies reported good cumulative survival rates, both for tooth-supported crowns (94.8% after 8 years [52]) and implant-supported crowns, made by CAD-CAM procedure following conventional impression (100% after 5 years [53]). A recent prospective study on implant-supported, single-unit monolithic restorations made of LS_2_ in a complete digital workflow has demonstrated survival rates of 100%, without any technical or biological complications, after 2 years of service [54]. Similarly, retrospective studies have shown that LS_2_ can yield satisfactory clinical performance with favourable survival rates and low incidence of mechanical failures, like debonding, fractures and chipping [15, 55–58].

As regards chairside procedures, monolithic LS_2_ crowns revealed a survival rate of 83.5% after 10 years of follow-up; the main complications were loss of retention, secondary caries and hypersensitivity [59].

In the last decade, LS_2_ has been proposed for producing full-contoured, monolithic SCs to be bonded to CAD-CAM zirconia full-arch frameworks supported by implants. In a mid-term study, such a restorative solution exhibited 100% survival rate, after 5 years of follow-up [60]. Recently, an in vitro study has suggested that LS_2_ crowns supported by ceramic-reinforced polyether ether ketone (PEEK) implant abutments may be an alternative to zirconia abutments with a titanium base for single-implant restorations in the anterior region [61].

Thanks to the high reliability of resin bond to glass-ceramics, LS_2_ clinical indications also include adhesively retained, tooth-supported restorations. In the anterior sites, in the authors’ and in other clinicians’ clinical experience, laminate veneers made of bilayered, hand-veneered LS_2_ are a likely choice, particularly when clinical performance and high esthetic results are expected [62]. Clinical and in vitro studies demonstrated that, in the presence of long teeth, margins positioned beyond the cemento-enamel junction (CEJ), large areas of exposed dentin or flexural tensile stresses due to high functional loads, laminate veneers are exposed to higher failure risks, being maximum enamel preservation and veneer mechanical resistance paramount success factors [63, 64]. Due to its mechanical properties, lithium disilicate can be considered a viable option to fabricate ceramic veneers in the presence of unfavorable biomechanical conditions; in fact, it was reported that more rigid ceramic materials exert a kind of shield effect onto underlying tooth structures, strengthening the restorative complex [65].

Since their introduction in 1991, all-ceramic, resin-bonded fixed dental prostheses (RBFDPs) have been increasingly utilized as minimally invasive restorations aimed at replacing one missing tooth in the anterior arch [66]. Although recording a high rate of early (1-year), unilateral retainer fractures in conventional, two retainers all-ceramic adhesive bridges, the authors noticed that the fractured, unilaterally supported restorations stayed in situ for 5 to 10 years [67–69]; for that reason, since 1997 cantilevered all-ceramic RBFDPs were proposed as a new conservative treatment modality for replacement of single anterior missing teeth, with minimal tooth preparation on the lingual side, just aimed at achieving a correct positioning during cementation [70]. Different materials have been proposed over the years, mainly, for their high strength, glass-infiltrated alumina ceramics [71] and densely sintered, bilayered zirconia, treated with a combination of moderate pressure air-abrasion and MDP, with promising medium-term outcomes [72–75]. Thanks to its advantageous optical properties and to its HF etching/silane bonding option, LS_2_ has also been proposed as an alternative material for such cantilevered restorations, showing comparably promising clinical results [76–78]. In a systematic review, cantilevered RBFDPs showed a lower failure rate than conventional, two-retainer, “Maryland bridge-style” ones, in which higher biomechanical stress arises for the different directions of forces acting on the adjacent supporting teeth during anterior guidance in protrusive and lateral mandibular movements [79]. In another recent review, an estimated 91.2% survival rate at 5 years was reported for all-ceramic RBFDPs, exhibiting higher debonding rate with zirconia resin-bonded restorations than with glass-ceramic ones; conversely a higher fracture rate was reported with glass-ceramics [80], even though higher level of evidence will be necessary to draw final long-term evaluations of all-ceramic RBFDPs clinical performances. RBFDPs are a suitable prosthetic solution as an alternative to implant-supported SCs, in the presence of anatomical impairment requiring costly and invasive surgical procedures, financial problems, young age of patients with congenitally or post-traumatically missing incisors; in any case, to limit the risks of mechanical failure or debonding, after an extensive esthetic, occlusal and technical evaluation of the case, a very careful treatment planning has to be defined prior to proceed with the operative phases.

In the posterior sites, LS_2_ can be successfully employed for resin-bonded single restorations, like inlays, onlays, non-retentive partial crowns and full coverage table-tops, in the monolithic form. The material offers undisputable advantages, like high fracture resistance, showed by high load-at-fracture values in table-tops/occlusal veneers, allowing reduced thickness of the restorations (1–1.5 mm), low wear and abrasive potential, adhesive bonding strength and high biocompatibility, properties that are very favourable when teeth are severely abraded or a heavy occlusal correction is needed (like in lateral post-orthodontic open bite) [10, 81–85]. These restorative solutions have shown favourable clinical outcomes in the most recent literature, even though with limited follow-up [86, 87]. A recent 3-years randomized, controlled prospective trial has shown that LS_2_ partial crowns can be used as successful restorative solutions for endodontically treated posterior teeth, with no significant differences between premolar or molars and with or without the use of fiber posts [88].

The utilization of LS_2_ for FDPs is a controversial topic: literature data is quite scant and not homogeneous, with a high variability of reported survival and success rates, ranging from rather poor clinical results [89–92] to acceptable long-term serviceability both in anterior and posterior sites, similar to metal-ceramics [93]. In the opinion of the authors, from a strictly clinical point of view, taking into account the cost/benefit ratio in terms of esthetic needs and structural resistance, the material of choice for 3- or 4-unit FDPs is still zirconia, in all of its different typologies.

#### Marginal accuracy and internal fit

Several studies evaluated the adaptation of lithium disilicate restorations, fabricated in both conventional and digital workflow. According to the most recent literature, there is no significant difference in terms of marginal accuracy between conventional and full-digital procedures for the fabrication of monolithic lithium disilicate crowns [94–96]. Moreover, some authors reported that hot-pressed LS_2_ crowns made from conventional impressions with polyvinylsiloxanes exhibit better fit than CAD-CAM digitally produced ones [97].

Furthermore, centralized milling production has been reported to result in better fit compared to chairside system; in the same study, occlusal internal adaptation was better in the conventionally manufactured crowns than in the digitally fabricated ones [95]. Conversely, other studies reported that marginal and internal fit of LS_2_ crowns were more accurate when using digital impression technique; in any case, whatever the workflow used, the adaptation was shown to be within clinical acceptability range [98–101].

To date, drawing univocal conclusions about adaptation accuracy of lithium disilicate restorations is not easy, due to the high number of variables involved in the final prosthetic fit, like digital impression system and technique, used material and fabrication procedure, so there is still a noticeable amount of controversial debate [3, 102]. As regards fabrication techniques, hot-pressed lithium disilicate is reported to offer better internal fit and mechanical performances compared to CAD-CAM pre-crystallized blocks, even if, also about this topic, further data will be necessary to definitely shed light on these aspects, due to the constant evolution and increasing quality of milling procedures and devices [103–108].

#### Zirconia reinforced-Lithium silicate ceramics (ZLS)

In the last years, the continuous research and progress in prosthetic material field for dental CAD-CAM applications has led to the introduction on the market of promising materials, the ZLS, thanks to an alternative strategy to enhance translucency: a glassy matrix, containing a homogeneous crystalline structure made of lithium silicate crystals, is reinforced with tetragonal zirconia fillers (about 10% by weight) allowing higher strength values than LS_2_ [109]. The higher mean translucency, together with proper biaxial flexural strength values, make such material a proper choice for minimally invasive, single tooth esthetic restorations, like inlays, onlays, partial crowns, veneers, anterior and posterior crowns, both tooth- and implant-supported [109, 110], also fulfilling the “no-prep, table-top” strategy [85]. The restorations show higher translucency and ease of intraoral polishing than both feldspathic and disilicate blocks, but, at the same time, exhibit high brittleness [110–112]. In case of a dark substrate, moreover, it has to be considered that the high translucency of the material requires adequate thickness (1.5–2.0 mm) in order to get a proper chromatic masking [113].

To date, as regards mechanical properties and clinical performances of ZLS, data are still limited, often controversial and short-term; these highly promising ceramics need further studies, both in vitro and in vivo, in order to precisely define physical-mechanical properties, clinical indications, limits and long-term performance of such restorations [114–117].

### Zirconia

#### Physico-chemical features

In the ceramic classification, zirconia (ZrO_2_) is a heterogenous, highly-resistant, polycrystalline ceramic, characterized by favourable mechanical properties (toughness: 5–10 MPa√m, flexural strength: 500–1200 MPa, Young’s modulus: 210 GPa) and good optical characteristics [118–121]; however, differently from glass-ceramics, it is not susceptible to conventional acid etching techniques and, consequently, does not take advantage of conventional adhesive bonding procedures [122].

Both in vitro and in vivo, it shows excellent biocompatibility, lower plaque retention than titanium and good radiopacity; moreover, it is not soluble in water and its susceptibility to corrosion in the oral environment is negligible [118–121]. Among the various metal-free, ceramic materials, after conventional finishing and polishing, monolithic zirconia exhibits the lowest wear behaviour towards opponent teeth [123].

#### Phase transformation toughening (PTT)

In dentistry, zirconia is usually considered an all-ceramic material but, from the physical-chemical point of view, it is a metal oxide with ceramic properties characterized by polymorphism and allotropy. In fact, it is present in nature with three different crystalline configurations at different temperatures: cubic (from the melting point at 2680 °C to 2370 °C), tetragonal (from 2370 °C to 1170 °C) and monoclinic (from 1170 °C to room temperature). These different allotropic states present with distinct mechanical and optical properties that can be exploited differently in Prosthodontics [118–121, 124].

Conventionally, zirconia is mainly used in its partially yttria-stabilized tetragonal phase (Y-TZP) as a prosthetic material for indirect restorations. Under the effect of mechanical, thermal and/or combined stresses, the adsorbed energy can break part of the atomic bonds of its polycrystalline structure turning such tetragonal crystals to a stabler monoclinic shape. This spontaneous and irreversible transformation is known as Phase Transformation Toughening (PTT) and shows a contemporary 4–5% increase in crystals volume, creating significant compressive stresses within the material [118–121, 124].

From the technological and prosthetic sides, the PTT has been advertised as a paramount advantage, since it allows a kind of self-repairability of zirconia; indeed, it permits to block or at least to hinder the propagation of micro-cracks and fractures within the material. In fact, the subsequent volumetric increment of the crystals generates comses within the material at the fracture tip, limiting crack propagation [118–121, 124–126]. It is worth noticing that at room temperature such transformation is irreversible and localized, centered at the stress bearing area (i.e. occlusal load area, traumatic impact zone, etc.): once the limiting action of the fracture propagation has occurred, in its monoclinic configuration zirconia is no longer able to limit cracks any further [119, 124, 126]. On the contrary, heating monoclinic zirconia again up to 900–1000 °C (for limited time according to manufacturers’ instructions), the PTT becomes reversible: by means of a process called “regeneration” or “annealing”, monoclinic crystals can be moved back to the tetragonal phase, causing the relaxation of compressive stresses within the material [125, 126]. After annealing, however, zirconia toughness tends to be reduced and, as regards the optical properties, a chromatic oversaturation can occur; consequently, thermal treatments at high temperature should be used carefully and only after potentially aggressive mechanical procedures (i.e. relevant occlusal grinding, polishing, etc.) [126–128].

In order to profit from the positive features of the PTT intraorally, during industrial manufacturing cubic and tetragonal zirconia are stabilized with metal oxides, just like yttrium, magnesium, cerium and lanthanum; the percentage of such dopants can vary according to manufacturing techniques and clinical use. These stabilizing oxides contribute to keep zirconia in its crystalline tetragonal phase also at room temperature in a thermodynamically metastable state, preventing the spontaneous transformation in the more stable monoclinic crystals. However, such dopant oxides can get lost after traumatic events, surface modifications (i.e. occlusal adjustments, grinding, polishing, etc.) and material aging [118–121, 124–127].

#### Low temperature degradation (LTD) and aging

In turn, the PTT is closely related to a negative phenomenon, the so-called “Low Temperature Degradation (LTD)”, responsible for zirconia aging. At room temperature, the material can undergo a spontaneous and irreversible transformation to the monoclinic phase, even in the absence of any mechanical stress. This phenomenon causes a worsening of mechanical properties, till the possible occurrence of spontaneous fractures [118–121, 124–127, 129, 130]. The LTD is a multifactorial phenomenon affected by several variables, such as crystals dimension, temperature, surface defects, manufacturing techniques, percentage and distribution of stabilizing oxides, mechanical stress and wetness; particularly, the last two factors can significantly accelerate zirconia aging. Although aging is considered a risk factor for mechanical failure, to date no univocal correlation has been evidenced between this phenomenon and the failures affecting zirconia during clinical service. Nonetheless, the LTD is known to cause a worsening of zirconia characteristics, contributing to the onset of micro-cracks, toughness reduction, increased wear, roughening and plaque accumulation, till a severe surface degradation, affecting both mechanical and optical properties [118–121, 125–127, 129, 130].

As reported in a recent in vitro study, monolithic tetragonal zirconia restorations can undergo hydrothermal degradation (i.e. aging) also after short observation times; however, such phenomenon does not reduce significantly the mechanical properties of tetragonal zirconia even in the presence of wide monoclinic transformed areas [126]. In the same research, the glassy layer used for glazing effect can act as a protective barrier against hydrothermal degradation; nonetheless, some restoration areas, particularly at the margins, can show absence of glazing protection and subsequently can be more susceptible to aging [126].

In vitro studies have clearly demonstrated that mechanical properties of zirconia, expressed by parameters like load-to-fracture values, are higher than those of LS_2_, which, from their part, are higher than those of ZLS; the number of fatigue loading cycles does not seem to affect the load-to-fracture of zirconia restorations [23].

#### Optical and mechanical properties

Laboratory investigations reported that monolithic zirconia restorations showed higher resistance to fracture than bilayered ones, even after mechanical cycling and aging [131–136]. Surface finishing techniques did not influence mechanical performance [132], neither did cementation techniques, particularly onto implants [137]; on the contrary, fracture resistance has been reported to be significantly influenced by preparation design [138, 139] and low temperature degradation [138], so it can be inferred that material and geometrical characteristics are crucial to optimize longevity of monolithic zirconia restorations [140]. The high mechanical reliability of zirconia has been confirmed by recent in vitro analyses, demonstrating that monolithic zirconia crowns with occlusal thickness of 0.5 mm exhibit sufficient fracture resistance to withstand occlusal loads in the molar regions [134, 135]. Moreover, increasing the content of yttrium oxide to improve the optical properties of zirconia can reduce mechanical properties after aging, although fracture resistance was reported to be higher than masticatory loads (3000 N) [141].

Zirconia is usually considered as an opaque restorative material with optical and esthetic properties less attractive than glassy ceramics, particularly in terms of translucency. By means of transillumination, it has been shown that tetragonal zirconia allows only about 25% of incident light to pass through; this characteristic can be advantageously used to mask dark substrates (i.e. metal posts/abutments, dark teeth, etc.) [126, 127, 142–144].

Recently, in order to enhance the esthetic properties of the material, translucent zirconia has been introduced in the market, characterized by the presence of 30–35% of cubic crystals. Besides the improved optical characteristics, in the presence of such cubic phase no hydrothermal degradation (i.e. aging) of this allotropic component is evidenced. However, apart from the better optical properties, the toughness of translucent zirconia is reduced, compared to tetragonal one, with values of flexural strength ranging between 500 and 900 MPa; as a consequence, translucent zirconia represents a suitable esthetic and mechanical compromise to be preferred in anterior areas up to the first premolars in its monolithic configuration [126, 142, 143]. As demonstrated by a recent investigation, the reduced mechanical properties of translucent zirconia are due to the dimensions and distribution of the crystals: in fact, cubic grains present with wider dimensions than tetragonal ones and segregate a higher amount of stabilizing oxides, making the tetragonal phase more prone to aging [126].

#### Manufacturing procedures

Although new additive technologies are emerging from the research on dental materials, to date, zirconia is still fabricated by CAD-CAM milling, according to two different production techniques: either soft machining of pre-sintered zirconia or hard machining of fully-sintered zirconia. Both procedures can be accomplished in industrial milling centers, in dental laboratories or by chairside devices [118–121, 124, 127].

Soft machining represents the most popular manufacturing technique and is based on milling of pre-sintered zirconia blanks fabricated by cold-isostatic pressing a mixture of zirconia powder, stabilizing oxides and binding agents (the latter removed during the pre-sintering process). With this technique, zirconia is highly homogenous and easier to mill, reducing production times, machinery wear and surface flaws; furthermore, soft machining generates negligible internal porosities (about 20–30 nm). The downside is that this process requires a 25% oversizing of the framework to be milled, since following sintering a linear shrinkage of the final volume occurs; as a consequence, although milling procedures are easier, soft machining requires a precise matching of CAD oversizing and material shrinking in order to avoid dimensional inaccuracies, particularly in the presence of complex framework geometry [118–121, 125, 127].

Viceversa, hard machining requires milling of fully-sintered zirconia blanks generally produced with hot isostatic pressing (HIP) at 1400°-1500 °C. This approach eliminates the problem of post-milling shrinkage, since neither oversizing nor sintering are necessary; however, hard machining needs longer milling times and more complex manufacturing, involving higher costs due to accelerated wear of production machinery and increased risks of attrition flaws. In addition, right after hard machining, zirconia frameworks can undergo a certain amount of monoclinic transformation phase due to mechanical stress, working burs friction and overheating subsequent to machining of the hard material [118–121, 125, 127].

Literature data are still controversial about which technique is the best, being the choice mainly guided by the operator preference, according to considerations related to shape, volume and complexity of the prosthetic geometry as well as time and cost of the milling procedures [118–121, 127].

High temperature and prolonged sintering time generate bigger zirconia crystals and the dimension of such grains significantly influences the mechanical properties of the material. In fact, the critical crystal dimension is about 1 mm: above this diameter, zirconia becomes spontaneously more susceptible to PTT, while under 0.2 mm such phenomenon does not occur and the toughness of the material decreases. Consequently, fabrication procedures (particularly sintering) significantly affect mechanical properties and stability of zirconia and have to be carefully checked during the whole manufacturing process [126, 127, 129, 130, 142].

In order to get a proper color of the restorations, specific metal oxides can be used as stains within the pre-sintering zirconia powder mixture or metallic salts can be infiltrated after milling; moreover, zirconia blanks are also available in multilayered color configurations. It has been clearly demonstrated that the coloring process does not influence mechanical properties of tetragonal zirconia, whilst uncertainty still remains regarding translucent cubic crystals [118–121, 125, 127, 129, 130].

Zirconia can be fabricated in monolithic or layered configurations. The monolithic material, not veneered with any ceramic layer, shows a less attractive esthetic appearance, but is not affected by the frequent cohesive fractures of the layering ceramics, known as “chipping” [134, 145].

To date, scientific evidences support the use of monolithic zirconia in posterior regions and in not esthetically relevant areas of the anterior arch (i.e. lingual tooth surfaces), while the use of layered restorations should be mainly addressed in highly esthetic zones [134, 145–149]. The minimum thickness suitable for monolithic Y-TZP restorations is 0.5 mm [134]; as regards layered prostheses, the total thickness ranges between 1.0 and 1.5 mm [134, 145–149]. In order to optimize mechanical resistance of layered restorations, it is paramount that veneering ceramics exhibit zirconia-compatible CTE [128, 150].

#### Marginal accuracy and internal fit

The accuracy of zirconia prostheses can be influenced by several factors, such as manufacturing, complexity of framework geometry (i.e. marginal finish line, span length, connectors dimension, etc.) and aging. The comparison of data regarding internal precision and marginal fit of zirconia is quite difficult, as literature data are heterogeneous and study designs are different for both laboratory and clinical investigations [119, 120, 127]. To date, it is possible to state that marginal precision of zirconia restorations is better than internal fit (probably because of the shape/size of the CAD-CAM milling burs) and that, in any case, precision values are well within the range of clinical acceptability reported in the specifications of the American Dental Association (ADA). Marginal gap values have been reported between 0 and 75 mm for SCs [151, 152] and 140 mm for FDPs, the latter showing an increasing proportional to framework span [119, 120, 127, 153].

As regards preparation geometry, the high stability and structural resistance of zirconia are compatible with both vertical and horizontal finish lines [124, 153].

#### Surface treatment and cementation

Due to the absence of any glassy matrix, zirconia is free from silica and, consequently, cannot be conditioned with conventional acid etching techniques, differently from glass-ceramics [119, 122]. Several surface treatments aimed at getting a reliable bond to the substrate have been reported in the literature but to date this topic is still controversial [154–163]. Aggressive sandblasting (i.e. 250 mm alumina particles at 0.4 MPa) can cause loss of the stabilizing oxides with a subsequent increased risk of accelerated PTT and aging of the material; as a consequence, it would be advisable to treat zirconia surfaces with milder sandblasting, using 110 mm alumina particles at 0.2 MPa. Such treatment can be advantageous for partially stabilized zirconia (PSZ) while it seems to weaken the fully stabilized material (FSZ) [155, 156, 158, 159, 163].

The use of coupling agents like silane can be adopted only after a tribochemical conditioning with silica-coated alumina particles or after infiltrating the zirconia surface with a thin layer of glassy ceramics [154, 155, 161]; however, the latter approach can determine the creation of excessive ceramic thickness and the effectiveness of adhesion between the glassy matrix and the polycrystalline network still remains unclear [154, 155, 158, 161].

The combination of mechanical and chemical treatments of zirconia surface was proved to offer the best results; particularly, the use of primers and adhesion promoting agents containing acidic monomers (10-MDP) can have a synergic effect with silane, improving the effectiveness of simplified adhesive techniques [155, 160–163].

On the basis of the physical-chemical properties of zirconia, in the presence of retentive preparation geometries and full coverage prostheses, conventional water-based luting agents (i.e. glass-ionomer and zinc phosphate cements) and hybrid cements (i.e. resin-modified glass-ionomer cements) can be considered a good choice for cementation. Otherwise, in the presence of partial coverage restorations, scarcely retentive preparation geometries (e.g. abutment teeth with reduced occluso-cervical dimension) and/or high masticatory loads, besides the above mentioned conditioning treatments of zirconia surface, it is possible to use conventional resin cement or simplified self-adhesive luting agents, so as to allow resin better adsorb, distribute occlusal forces and withstand possible micro-cracks on the inner surface of the restorations [155, 158, 162].

#### Clinical indication and performances

From a clinical point of view, in the last decades zirconia has more and more gained ground in the realm of metal-free, mainly utilized to restore both natural teeth and osseointegrated implants with SCs and short- and medium-span FDPs up to 5 elements [134, 145, 146, 148, 149, 164, 165]. As regards FDPs, besides the high mechanical properties of the material, fracture resistance and clinical performance are also strongly related to a proper framework architecture. In case of bilayered FDPs, in particular, an “anatomic” design has to be performed, ensuring proper support and thickness to the veneering; moreover, connectors are to be designed with adequate dimensions (minimum area of section: 9, 15 and 25 mm^2^ for 3-, 4- and 5-unit FDPs respectively) and with rounded interdental embrasures, in order to avoid sharp angles that can contribute to generate risky stress concentration [146]. The presence of an adequate occlusal support is a relevant factor in maintaining an efficient chewing [166]; consequently, due to the absence of veneering ceramics that could be subjected to wear over time, monolithic restorations could be helpful in keeping occlusal stability during clinical service, particularly in the presence of discrepancies in occlusal contact patterns that could influence the onset of temporo-mandibular disorders [167].

Recently, clinical investigations regarding tooth- and implant-supported full-arch restorations have been published [165]. Although short- and medium-term results were encouraging with 94.8% success rate after 3 years of clinical service for monolithic full-arch bridges [145], it is worth noticing that a systematic review of the literature has reported 5-year complication rates of 27.6 and 30.5%, respectively for tooth-supported and implant-supported full-arch restorations [168]. Moreover, layered restorations showed 5-year success rates significantly lower than monolithic prostheses (i.e. 60.4% vs 90.9%) [169]. Consequently, the use of full-arch, extended zirconia restorations should always be carefully evaluated and further long-term clinical studies are necessary to validate the effectiveness of their serviceability.

As regards zirconia implants, the literature reports controversial, short-term and mainly anecdotal data [165, 170–174]. A recent systematic review with meta-analysis has evidenced similar potentialities of hard- and soft-tissue integration between zirconia and titanium implants, although with a slower initial osseointegration process detected in zirconia ones. In any case, the use of the latter should be cautiously evaluated, until more light is shed on long term outcomes and, particularly, on the possible mechanical complications. Viceversa, zirconia abutments are to be considered widely validated today in the esthetic sites, where the clear color of zirconia contributes to achieve a natural aspect of peri-implant soft tissues, particularly when they are quite thin [127, 148, 165, 172, 173]. A retrospective clinical study on a relevant number of ceramic abutments reported that internal zirconia implant connections are much more prone to mechanical complications (i.e. unscrewing, fractures, etc.) than hybrid connections with zirconia abutments cemented onto titanium bases; moreover, the same investigation reported that the distance between the implant/abutment connection and the occlusal plane can significantly influence the onset of bending moments that can be detrimental for the long-term prognosis of metal-free restorations [172].

## Conclusions

At the moment, it can be stated that silicate- and zirconia-based ceramics are amongst the most versatile metal-free materials available for the “digital prosthodontic environment”. In the last years, an increasing amount of available in vitro and in vivo data is shedding precious light on the outline of guidelines for a restorative rational use, focused on specific materials advantages and limitations, taking into account mechanical, optical and biological properties in the light of a widespread clinical experience (Table 1). In the meanwhile, the world of industry is intensively working on new strategies aimed at further enhancing microstructural characteristics of these materials, together with the introduction of new production technologies, mainly based on additive processes.

**Table 1:** Lithium disilicate and zirconia: pros and cons

Lithium disilicate	
Pros	Cons
• excellent optical characteristics and good mechanical properties • clinical versatility • biocompatibility • favourable abrasiveness • marginal accuracy and internal fit • high strength of adhesion to the substrate • monolithic and layered	• glaze coating and fluorapatite ceramic veneering can increase wear • critical to adjust intraorally • chipping of the veneering ceramics
Zirconia	
Pros	Cons
• excellent mechanical characteristics and good optical properties • excellent biocompatibility and low plaque retention • favourable wear behaviour • implant abutments for esthetic sites • crack-hindering potential (through PTT) • marginal accuracy and internal fit • monolithic and layered	• opacity • unetchable with conventional methods • low temperature degradation and aging • critical to adjust intraorally • glaze coating and fluorapatite ceramic veneering can increase wear • chipping of the veneering ceramics

## Data Availability

Not applicable.

## Abbreviations

10-MDP: 10-Methacryloyloxydecyl-Dihydrogen-Phosphate
ADA: American Dental Association
Ca_5_(PO_4_)_3_F: Fluorapatite crystals
CAD-CAM: Computer-aided design/computer-aided manufacturing
CEJ: Cemento-enamel junction
CTE: Coefficient of thermal expansion
FDPs: Fixed dental prostheses
FSZ: Fully stabilized zirconia
HF: Hydrofluoric acid
HIP: Hot isostatic pressing
KIC: Fracture toughness
Li_2_Si_2_O_5_: Lithium disilicate crystal nuclei
Li_2_SiO_3_: Metasilicates
LS_2_: Lithium disilicate
LTD: Low Temperature Degradation
PEEK: Polyether ether ketone
PSZ: Partially stabilized zirconia
PTT: Phase Transformation Toughening
RBFDPs: Resin-bonded fixed dental prostheses
SCs: Single crowns
SEM: Scanning electron microscope
Y-TZP: Yttria stabilized tetragonal zirconia
ZLS: Zirconia reinforced-Lithium Silicate ceramics
ZrO_2_: Zirconia
